# Emerging Concern for Silver Nanoparticle Resistance in *Acinetobacter baumannii* and Other Bacteria

**DOI:** 10.3389/fmicb.2021.652863

**Published:** 2021-04-16

**Authors:** Oliver McNeilly, Riti Mann, Mohammad Hamidian, Cindy Gunawan

**Affiliations:** ^1^iThree Institute, University of Technology Sydney, Ultimo, NSW, Australia; ^2^School of Chemical Engineering, University of New South Wales, Sydney, NSW, Australia

**Keywords:** antibiotic resistance, silver nanoparticles, *Acinetobacter baumannii*, silver resistance, co-selection

## Abstract

The misuse of antibiotics combined with a lack of newly developed ones is the main contributors to the current antibiotic resistance crisis. There is a dire need for new and alternative antibacterial options and nanotechnology could be a solution. Metal-based nanoparticles, particularly silver nanoparticles (NAg), have garnered widespread popularity due to their unique physicochemical properties and broad-spectrum antibacterial activity. Consequently, NAg has seen extensive incorporation in many types of products across the healthcare and consumer market. Despite clear evidence of the strong antibacterial efficacy of NAg, studies have raised concerns over the development of silver-resistant bacteria. Resistance to cationic silver (Ag^+^) has been recognised for many years, but it has recently been found that bacterial resistance to NAg is also possible. It is also understood that exposure of bacteria to toxic heavy metals like silver can induce the emergence of antibiotic resistance through the process of co-selection. *Acinetobacter baumannii* is a Gram-negative coccobacillus and opportunistic nosocomial bacterial pathogen. It was recently listed as the “number one” critical level priority pathogen because of the significant rise of antibiotic resistance in this species. NAg has proven bactericidal activity towards *A. baumannii*, even against strains that display multi-drug resistance. However, despite ample evidence of heavy metal (including silver; Ag^+^) resistance in this bacterium, combined with reports of heavy metal-driven co-selection of antibiotic resistance, little research has been dedicated to assessing the potential for NAg resistance development in *A. baumannii*. This is worrisome, as the increasingly indiscriminate use of NAg could promote the development of silver resistance in this species, like what has occurred with antibiotics.

## Introduction

The WHO has acknowledged that, alongside climate change and non-communicable disease, bacterial antibiotic resistance represents one of the most important crises to human health today (Cassini et al., 2019). It is projected that over 33,000 people in Europe alone die annually from resistant bacterial-related infections, making it a near equal health burden to influenza, HIV, and tuberculosis combined (Cassini et al., 2018, 2019). In 2014, it was estimated that infection from antibiotic-resistant bacteria in the United States resulted in a loss of over $20 billion in direct economic costs, and $35 billion through decline in societal productivity (Golkar et al., 2014; Zhen et al., 2019). The leading cause of nosocomial infections globally is due to a league of bacteria which readily develop drug resistance, collectively referred to as the ESKAPE pathogens (Rice, 2008; Santajit and Indrawattana, 2016). This group includes: *Enterococcus faecium*, *Staphylococcus aureus*, *Klebsiella pneumoniae*, *Acinetobacter baumannii*, *Pseudomonas aeruginosa*, and *Enterobacter* spp. The ESKAPE organisms represent the model archetype of virulent and adaptive bacterial organisms, as they frequently cause severe and chronic disease and ‘escape’ the activity of antibiotics (Santajit and Indrawattana, 2016).

Of this group, *A. baumannii* has attracted significant attention over the last two decades due to the rapid onset of antibiotic resistance and worldwide spread of this species (Howard et al., 2012). It is a Gram-negative, strictly aerobic coccobacilli and opportunistic bacterial pathogen that is generally associated with nosocomial infections, causing a range of nonspecific infections including pneumonia, soft tissue necrosis, and sepsis (Heritier et al., 2006; Alsan and Klompas, 2010; Al-Anazi and Al-Jasser, 2014; Chen et al., 2020). This bacterium became important throughout the 2001–2007 Iraqi-Afghan desert conflicts. Numerous medical and epidemiological reports documented a high incidence of multi-drug resistant *A. baumannii* infections among injured British and United States soldiers, with one report stating that 37% of the isolates was carbapenem-resistant (Alsan and Klompas, 2010; Howard et al., 2012; Hamidian and Nigro, 2019). International travel, including transportation of returning soldiers, is thought to be the main contributing factor in the global dissemination of resistance in *A. baumannii* (Peleg et al., 2008). *A. baumannii* is naturally resistant to desiccation and is primarily isolated on medical equipment in hospitals, rather than in nature, and this frequently results in the infection of patients needing treatment with invasive apparatuses (Towner, 2009). The recent COVID-19 pandemic has led to a significant surge in hospital and intensive-care unit (ICU) admissions. There have been numerous challenges in ensuring that adequate personal protective equipment (PPE) is available for medical staff and that routine sterility management practices are maintained in COVID-19 dedicated hospitals (Jain, 2020; Gottesman et al., 2021). Studies have reported increasing incidences of drug-resistant bacterial co-infections in COVID-19 patients, most often due to cross-contamination from other patients/staff and unsterile equipment (Chen et al., 2020; Sharifipour et al., 2020). Many of these incidences have included outbreaks of *A. baumannii* co-infections, particularly in ICUs, several of which have been identified as carbapenem-resistant (Perez et al., 2020; Sharifipour et al., 2020; Gottesman et al., 2021). These cases of *A. baumannii* secondary infections throughout COVID-19 dedicated hospitals has not only further burdened already pressured medical sectors around the globe, but could also inevitably accelerate the propagation and spread of antibiotic-resistant *A. baumannii* and other priority bacterial species (Clancy et al., 2020; Hsu, 2020).

The rapid emergence of drug resistance in *A. baumannii* has resulted from its ability to acquire resistance genes and adapt to environmental selective pressures (Alsan and Klompas, 2010). Consequently, this had led to the generation of multi-, extensive-, and pan-drug-resistant strains of *A. baumannii*, the bulk of which belong to two clonal lineages, namely global clone 1 (GC1) and global clone 2 (GC2; Gonzalez-Villoria and Valverde-Garduno, 2016; Hamidian and Hall, 2018; Hamidian and Nigro, 2019). Resistance development in *A. baumannii* is generally accomplished through three mechanisms: (1) acquisition of resistance genes (mainly *via* bacteria-to-bacteria horizontal gene transfer), which most often encode drug-inactivating enzymes, such as OXA-type β-lactamases (e.g., OXA-23) which hydrolyses carbapenems; (2) insertion sequence (IS)-mediated activation of resistance genes, e.g., insertion of ISAba1 upstream of the intrinsic *A. baumannii* gene *ampC* provides it with a strong promoter and results in resistance to 3rd generation cephalosporins; and (3) genetic mutation, e.g., *gyrA* and *parC* mutations alter DNA gyrase and topoisomerase IV active sites and blocks the action of quinolones (Heritier et al., 2006; Hujer et al., 2006; Asif et al., 2018). This organism was introduced as an ESKAPE member in 2009, and in 2017, the WHO and the Centers for Disease Control and Prevention (CDC) declared carbapenem-resistant *A. baumannii* as the “number one” critical priority antibiotic-resistant pathogen among a list of 12 bacteria requiring urgent antibacterial research and development (Gonzalez-Villoria and Valverde-Garduno, 2016; World Health Organization, 2017; Centers for Disease Control and Prevention, 2019). Ultimately, antibiotic resistance in *A. baumannii* and the other priority ESKAPE pathogens highlights the need for immediate action of establishing new and alternative antibacterial agents to curb the threat of infection caused by these organisms (Rice, 2008).

But, the current rate at which new drugs are being developed is very slow. Most major pharmaceutical companies have withdrawn from financially supporting the research and development of new antibiotics due to a lack of government incentives for these high risk investments (Fair and Tor, 2014; Michael et al., 2014; Ventola, 2015; Zheng et al., 2018). Naturally, the need for antibiotic substitutes is dire, and nanotechnology has proven to offer effective alternatives (Howard et al., 2012). Nanoparticles are organic (i.e., carbon-sources) or inorganic (i.e., metals) based materials, ranging in 1–100 nm in size (Silva, 2004). Silver nanoparticles (nanosilver; herein after referred to as NAg) are currently the most widely produced nanoparticle, attributed to its unique physicochemical characteristics and multifaceted antimicrobial mechanisms (Silva, 2004; Mody et al., 2010). Many studies have demonstrated the antimicrobial efficacy of NAg against many viral, fungal, parasitic, and bacterial organisms (Rai et al., 2012; Ge et al., 2014). The healthcare sector is one of the largest markets for NAg, with the nanoparticle being used as a coating agent in medical devices, such as intravenous catheters, wound dressings, and organ/dental implants to inhibit bacterial colonisation (Khan et al., 2017). Worryingly, NAg has also been incorporated into many consumer products, and can, for example, be found in household appliances, textiles and clothing, cosmetics, childcare products, and food packaging and containers (Schäfer et al., 2011; Khan et al., 2017).

The widespread use of NAg has triggered concerns for the development of silver-resistant bacteria, diverging from the once commonly held perception that bacteria could not develop resistance to the nanoparticle (or silver in general) due to its complex antibacterial mechanisms (Rai et al., 2009; Gunawan et al., 2017). Over several years, a growing number of studies have been published describing the phenomenon of resistance in bacterial species in response to different forms of silver agents, including NAg. Silver resistance has been reported in *A. baumannii* and many other important pathogenic bacteria (Gupta et al., 1999; Gunawan et al., 2013; Muller and Merrett, 2014; Panáček et al., 2018; Hosny et al., 2019; Valentin et al., 2020).

In this paper, we highlight the antibacterial actions of NAg with its multi-targeting toxicity on *A. baumannii* and other bacteria. We also outline current knowledge on the adaptive ability of *A. baumannii*, and other significant bacterial species, to NAg and other silver agents. It is important that we understand the applicability, as well as the equally important long-term risks of the nanoparticle as a crucial alternative antimicrobial. This review also describes the emerging phenomenon of the metal-driven co-selection of antibiotic resistance, including silver, to further stress the issue of overexposing bacteria to toxic heavy metals.

## Physicochemical Factors and Antibacterial Properties of NAg

Silver nanoparticle has a number of physicochemical characteristics that affect its microbiological activity and overall stability (Rai et al., 2012). The nanoparticle exhibits distinct multi-targeting bactericidal mechanisms which are unique from common antibiotics and evidently underlines why NAg has become a popular alternative antibacterial agent (Rai et al., 2012; Yun’an Qing et al., 2018).

### Size, Shape, and Surface Properties of NAg

The physicochemical characteristics of NAg directly influence its antibacterial activity. The nanoparticles range from 1 to 100 nm size, and with their high surface-area-to-volume (SAV) ratios, each particle contains approximately 10,000–15,000 silver atoms, rendering them highly reactive (Morones et al., 2005; Zhang et al., 2016). Studies have shown that smaller particles have a higher SAV ratio and are generally associated with better physical nanoparticle-to-cell contact, and this allows for greater adherence to the bacterial surface which enhances their antibacterial activity (Morones et al., 2005; Zhang et al., 2016; Syafiuddin et al., 2017). NAg particles can be synthesised into different shapes, with the most common including spheres, truncated triangles, and rods/cylinders (Pal et al., 2007; Rai et al., 2009, 2012). A comparative study by Pal et al. (2007) found that truncated triangular shaped nanoparticles were most effective against *Escherichia coli* when compared to spherical and rod-shaped particles. The triangular shape was thought to improve reactivity of the nanoparticle due to the presence of unique active facets, which are associated with a greater concentration of silver atoms. It was also speculated that this shape enhanced particle adherence onto the bacterial surface, resulting in more extensive membrane damage and subsequent cell killing (Pal et al., 2007; Ge et al., 2014).

The size and shape of NAg determine, at least in part, the particle concentration required for effective toxicity. For example, higher concentrations of rod-shaped NAg particles at 10–100 nm (lower SAV ratio) are needed to display comparable antibacterial activity with those of truncated triangles at 1–10 nm (higher SAV ratio; Morones et al., 2005). Moreover, higher concentrations of NAg are generally required to inhibit Gram-positive bacteria in comparison to Gram-negatives (Shrivastava et al., 2007; Yun’an Qing et al., 2018). This is thought to associate with the presence of the thicker outermost peptidoglycan cell wall layer (30–100 nm) in Gram-positive bacteria, compared to those in Gram-negative bacteria (thin 2–8 nm peptidoglycan layer located between the outer and inner lipid membranes; Shrivastava et al., 2007; Silhavy et al., 2010; Yun’an Qing et al., 2018).

Another important characteristic of NAg that affects its toxicity is the presence of functional groups on the nanoparticle surface which, in many cases, act as stabilisers (to prevent aggregation) and influence the nanoparticle net surface charge (El Badawy et al., 2010; Zhang et al., 2016; Fahmy et al., 2019). Measured as zeta potential (ζ), NAg can have a positive or negative net surface charge depending on the surface functional groups (a nominally high ζ potential reflects good colloidal stability), which affects the particle electrostatic attraction with the bacterial surface (El Badawy et al., 2010). Under normal physiological conditions, the bacterial envelope has a net negative charge. The envelope itself is assembled with various biomolecules, e.g., proteins, sugars, and phospholipids, which contain many negatively charged functional groups, including carboxyl and phosphate groups (Silhavy et al., 2010). Studies have shown that nanoparticles with a highly positive ζ exhibit a greater extent of antibacterial effect than those with negative ζ, most likely due to the particle-to-bacterial envelope electrostatic interactions for the former (El Badawy et al., 2010; Rai et al., 2012; Yun’an Qing et al., 2018). This attraction allows for greater adherence and accumulation of NAg to the bacterial surface, with some studies hypothesising that this can induce neutralisation of the cell membrane and lead to the loss of its selective permeability (Morones et al., 2005; Tang and Zheng, 2018). El Badawy et al. (2010), for example, studied NAg with different coatings and observed the highest extent of bacterial surface interaction with a (branched) polyethyleneimine (BPEI)-coated nanoparticle type. This BPEI-NAg exhibited the most positive ζ [compared to negatively charged polyvinylpyrrolidone (PVP)-NAg and citrate-NAg], which was thought to interact closely with the bacterium (*Bacillus* sp.) due to electrostatic attractions (El Badawy et al., 2010; Tang and Zheng, 2018).

### General Antibacterial Mechanisms of NAg (Cell Surface)

**Figure 1:**
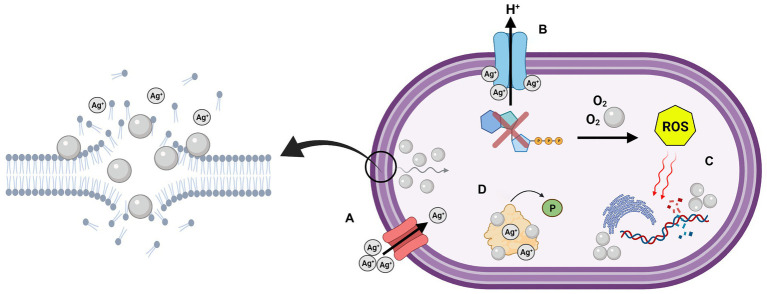
Graphical depiction of the multi-target antibacterial mechanisms of silver nanoparticles (NAg) on the cell surface and cell cytoplasm. **(A)** Adhesion and “pitting” of the cell membrane, and subsequent internalisation of NAg, along with Ag^+^ passage through outer membrane porin (OMP) channels, **(B)** uncoupling of respiratory chain by Ag^+^, **(C)** damage to biomolecules by reactive oxygen species (ROS) and intracellular NAg, and **(D)** disruption of cell signalling through protein dephosphorylation. Created in BioRender. Adapted from Dakal et al. (2016).

The antibacterial activity of NAg has been studied quite extensively (Rai et al., 2012). Four main antibacterial modes have been proposed: (A) bacterial envelope adhesion of the nanoparticle, resulting in envelope damage and cellular penetration, (B) uncoupling of the respiratory chain, (C) damage to cellular biomolecules and function, and (D) disruption of cell signalling (Figure 1; Dakal et al., 2016; Duval et al., 2019). As briefly discussed in “Size, Shape, and Surface Properties of NAg” section, direct physical contact of NAg with the bacterial surface is one of the initial mechanisms of its antibacterial function (Pal et al., 2007; Ge et al., 2014; Dakal et al., 2016). In Gram-negative bacteria, the ‘sticking’ of NAg to the outer membrane rapidly destabilises the membrane, allowing smaller-sized particles to enter the cell. This coincides with the formation of electron dense “pits” in the thin peptidoglycan layer, which enables the nanoparticle to target the inner membrane (Santajit and Indrawattana, 2016; Zhang et al., 2016; Tang and Zheng, 2018). The pitting effect is generally slower in Gram-positives, which is most likely due to the thicker outermost peptidoglycan cell wall layer (Dakal et al., 2016; Pazos-Ortiz et al., 2017; Tang and Zheng, 2018).

In an aqueous environment, NAg can interact with molecular oxygen (O_2_), which leads to oxidative dissolution. This causes leaching of silver ions (Ag^+^) from the nanoparticle, which are crucial to the overall antibacterial activity of NAg (Marambio-Jones and Hoek, 2010; Tang and Zheng, 2018). The morphology of NAg has been found to affect the extent and rate of Ag^+^ release, for example, smaller sized nanoparticles (with a higher SAV ratio) have been associated with a greater rate of ion leaching compared to larger particles (Xiu et al., 2012). Ag^+^ leached during the dissolution process are also thought to damage the bacterial membrane and membrane-bound proteins (Dakal et al., 2016; Yun’an Qing et al., 2018). Ag^+^ acts as a soft acid and has a high affinity for electron donor groups in amino acid constituents of structural proteins and enzymes, in particular, thiol (-S^−^) groups that are present in the amino acids cysteine and methionine, as well as amine (NH_x_) groups in histidine (−NH^+^), arginine (−NH_2_^+^), and lysine (−NH_3_^+^; Lara et al., 2010; Marambio-Jones and Hoek, 2010; Rai et al., 2012; Yun’an Qing et al., 2018). Ag^+^ has been found to bind to membrane-bound transport proteins in bacteria, subsequently inhibiting the proton motive force which disrupts the in-and-out transport of protons, as well as phosphate, necessary for ATP synthesis (Lok et al., 2006). Comparable to that of NAg, studies have also reported less occurrences of Ag^+^ penetration in Gram-positive bacteria when compared to Gram-negative bacteria. The cations are thought to be sequestrated within the thicker negatively-charged peptidoglycan layer of the former, rendering them more tolerant to Ag^+^ (Dakal et al., 2016; Vila Domínguez et al., 2020). However, evidence suggests that Ag^+^ is more potent than NAg against both Gram-positive and Gram-negative bacteria at equivalent silver concentrations (Li et al., 2017; Kędziora et al., 2018). In Gram-negative bacteria, this could be due to the presence of molecular transport channel proteins, e.g., outer membrane porins (OMPs), which facilitate transmembrane diffusion of ions, and in this case, Ag^+^, into the cytoplasm (Nikaido, 1994; Lok et al., 2006; Radzig et al., 2013; Kędziora et al., 2018).

The silver-induced ‘pitting’ effect on the cell wall along with altered membrane permeability inevitably allows smaller NAg particles and Ag^+^ to penetrate through the cell envelope and into the cytoplasm, while larger nanoparticles remain outside the cell (Sánchez-López et al., 2020). Studies have also hypothesised a Trojan horse-type mechanism, whereby the nanoparticles are absorbed intracellularly and undergo further leaching of Ag^+^, increasing local ion concentrations (Lemire et al., 2013; Różalska et al., 2018). Taken together, these mechanisms provide access for NAg/Ag^+^ to target intracellular structures and biomolecules, as described in the next section (Marambio-Jones and Hoek, 2010; Dakal et al., 2016; Zhang et al., 2016).

### General Antibacterial Mechanisms of NAg (Intracellular)

As discussed in “General Antibacterial Mechanisms of NAg (Cell surface)” section, Ag^+^ ions leached from NAg have been indicated to form complexes with electron donor groups, among these including thiol and amine groups present in structural proteins and enzymes (Lara et al., 2010). In addition to the disruption of membrane-bound transport proteins, studies have also reported inhibition of respiratory chain enzymes (e.g., NADH dehydrogenase) embedded in the inner membrane, which has been correlated to the complexing activity of Ag^+^ (Lara et al., 2010; Marambio-Jones and Hoek, 2010; Berrisford et al., 2016). The latter is thought to result in the uncoupling of electron transport necessary for oxidative phosphorylation, and in turn, inhibits the bacteria respiration process and synthesis of ATP (Holt and Bard, 2005; Marambio-Jones and Hoek, 2010). Furthermore, the disruption of the respiratory chain has been hypothesised to cause electron leakage, which reduces the presence of molecular O_2_ in the cytoplasm and leads to an elevated presence of NAg-induced reactive oxygen species (ROS), including superoxide (O_2_^•−^), in bacteria (Holt and Bard, 2005; Marambio-Jones and Hoek, 2010; Dakal et al., 2016). Studies have also suggested that Ag^+^ (and O_2_^•−^ radicals) can target iron-sulphur (containing thiol groups) clusters in proteins, releasing Fenton-active free Fe^2+^ ions which can react with cellular hydrogen peroxide (H_2_O_2_) and consequently generate highly reactive hydroxyl radicals (OH^•^; Xu et al., 2012; Ezraty et al., 2017). Excessively generated ROS can target cellular biomolecules and lead to oxidative stress. This can cause DNA damage and inhibition of replication, disruption of tRNA-30S ribosomal complexes involved in protein synthesis, as well as damage of proteins, e.g., *via* carbonylation, and lipids, e.g., *via* peroxidation, which has been observed for membrane phospholipids (Xiu et al., 2012; Zhao and Drlica, 2014; Kędziora et al., 2018). Interestingly, NAg has been shown to be less effective against strictly anaerobic bacteria when compared to aerobic bacteria, and this is indeed in agreement with the established role of O_2_ in radical oxygen generation, and, as mentioned earlier, in the oxidative leaching of Ag^+^ from the nanoparticle, both extracellularly and intracellularly (Xiu et al., 2012; Zhao and Drlica, 2014; Kędziora et al., 2018). Research inquiries have also suggested that NAg/Ag^+^ inhibit the activity of cellular antioxidants, such as glutathione (GSH) in Gram-negative bacteria (Marambio-Jones and Hoek, 2010). Note that, under normal conditions, ROS, including O_2_^•−^ and H_2_O_2_, are naturally generated in cells as by-products of respiration, and are neutralised by antioxidant systems when they exceed the homeostatic threshold (Ray et al., 2012; Gunawan et al., 2020). GSH neutralises ROS to non-toxic compounds, e.g., water, and in the process, GSH is oxidised to glutathione disulfide (GSSG; Marambio-Jones and Hoek, 2010; Dakal et al., 2016; Slavin et al., 2017). It is thought that NAg directly targets GSH, a glycine-cysteine-glutamic acid tripeptide (containing thiol groups), or alternatively, denatures the GSH reductase enzyme, which catalyses the GSSG-to-GSH recycling reaction (Slavin et al., 2017). For example, Singh et al. (2018) reported a decrease in the cellular presence of GSH (as well as cysteine) in *A. baumannii* with increasing NAg concentrations.

Silver nanoparticles and Ag^+^ have been indicated to interact with nucleotides (nucleoside-phosphate groups) in DNA, intercalating between the base pairs and binding to the nucleoside structural unit (Slavin et al., 2017). Some reports have found that Ag^+^ causes DNA condensation in both Gram-negative and Gram-positive bacteria, which is further linked to the observed inhibition of DNA replication (Feng et al., 2000; Guzman et al., 2012). Most reports have suggested that this condensation only occurs in the presence of Ag^+^, while the nanoparticle is associated with DNA fragmentation (an outcome of hydrogen-bond disruption between nucleotides; Feng et al., 2000; Rai et al., 2012; Slavin et al., 2017). NAg and Ag^+^ have also been found to modulate protein phosphorylation, which affects bacterial signalling pathways (Kirstein and Turgay, 2005; Shrivastava et al., 2007; Dakal et al., 2016). Protein phosphorylation acts as an essential signal relay mechanism in bacteria (and other domains of life) as it manages the “on and off” switching of proteins (Garcia-Garcia et al., 2016). Due to the high affinity of NAg/Ag^+^ for negatively charged phosphate groups, studies have shown that phosphorylated amino acid residues (e.g., tyrosine) in proteins can be dephosphorylated by both forms of silver, which consequently changes protein conformity and disrupts cell function (Shrivastava et al., 2007; Dakal et al., 2016).

## Combatting Antibiotic Resistance with NAg

A major factor to the increasing use of NAg is its proven efficacy against bacteria like *A. baumannii*, which can readily display resistance against antibiotics (Lara et al., 2010; Rai et al., 2012). The antibacterial effect of NAg is in general unaffected by antibiotic resistance mechanisms because of the nanoparticles’ multi-targeting mechanisms (Rai et al., 2012). Moreover, NAg has shown promising synergy with conventional antibiotics, exhibiting enhanced toxicity when compared to NAg or antibiotics alone, even against multi-resistant bacterial species (Baptista et al., 2018). The nanoparticle could also provide a solution to the current challenge of managing chronic bacterial infections, which are often associated with the colonisation of naturally resilient biofilms (Radzig et al., 2013).

### Effect of NAg on *A. baumannii* and Other Drug-Resistant Bacteria

Several reports have described the antibacterial effects of NAg on susceptible and multi-drug-resistant (MDR) *A. baumannii* (Table 1) and other various Gram-negative and Gram-positive bacteria, highlighting little difference in the nanoparticle toxicity on wild-type (or non-resistant) strains when compared to resistant strains (Lara et al., 2010; Rai et al., 2012). Many researchers have compared the bactericidal activity of NAg on a variety of bacterial species, including several ESKAPE members, such as methicillin-resistant *S. aureus* (MRSA), ampicillin-resistant *E. coli*, MDR *P. aeruginosa*, ampicillin-resistant *K. pneumoniae*, and *Salmonella typhi* (Percival et al., 2007; Shrivastava et al., 2007; Lara et al., 2010; Hamida et al., 2020). Each study indicated that NAg toxicity was independent of any of the antibiotic resistance traits in these bacteria, which is thought to be due to the multi-target mechanisms of the nanoparticle. Research on the activity of NAg on *A. baumannii* has also (mostly) shown comparable efficacy of the nanoparticle against wild-type and resistant strains (Łysakowska et al., 2015; Silva Santos et al., 2016; Chen et al., 2019a; Vila Domínguez et al., 2020). In contrast, however, Łysakowska et al. (2015), when assessing NAg activity on several wild-type and MDR *A. baumannii* strains, found that on average, the resistant types were less sensitive [minimum inhibition concentration (MIC) = 0.78 μg/ml] to NAg than the wild-type strains (MIC = 0.39 μg/ml). Although a minor difference in efficacy was observed, the team hypothesised that there was caused by ‘partial’ NAg cross-resistance in the MDR strains due to the presence of in-built antibiotic resistance mechanisms, e.g., efflux pumps (Łysakowska et al., 2015). Cavassin et al. (2015) studied NAg with various surface coatings on carbapenem and polymyxin-B-resistant *A. baumannii*. Similar to Łysakowska et al., the work also found that the resistant *A. baumannii* were less sensitive to the nanoparticle than the wild-type strains (Cavassin et al., 2015; Łysakowska et al., 2015). Further, Cavassin et al. found little difference in the nanoparticle toxicity between the citrate‐ (highly negative ζ) and chitosan‐ (highly positive ζ) coated NAg particles (Cavassin et al., 2015; Łysakowska et al., 2015). Both types were equally more effective against resistant *A. baumannii* and other tested species when compared to the other coating type (PVA-coated; ζ potential was close to zero causing particle aggregation; El Badawy et al., 2010; Cavassin et al., 2015). This is in contrast to the observations by El Badawy et al. (2010) who noted that positively-charged nanoparticle coatings were most often correlated with a higher extent of toxicity due to closer attraction with the negatively-charged bacterial cell surface.

**Table 1:** Examples of several investigations on the antibacterial activity of NAg against various multi-drug-resistant (MDR) and non-MDR *Acinetobacter baumannii* strains.

*A. baumannii* strain	NAg MIC^1^	NAg size (nm)^2^	Reference
*A. baumannii* (carbapenem‐ and PMB-resistant) *A. baumannii* (carbapenem‐ and PMB-susceptible)	3.4 μg/ml (Citrate-NAg) 6.7 μg/ml (Chitosan-NAg) 13.5–≥54 μg/ml (PVA-NAg) 1.6–3.4 μg/ml (Citrate-NAg) 1.6–3.4 μg/ml (Chitosan-NAg) 6.7–≥54 μg/ml (PVA-NAg)	40 25 10	Cavassin et al., 2015
*A. baumannii* (MDR)	≤10 μg/ml	5–10	Chen et al., 2019a
*A. baumannii* ATCC 19606 *Acinetobacter* spp. (clinical isolates)	0.78 μg/ml 0.39–0.78 μg/ml	2–5	Łysakowska et al., 2015
*A. baumannii* aba1604 (carbapenem-resistant)	2.5 μg/ml	8.4	Wan et al., 2016
*A. baumannii* AIIMS 7 (planktonic) *A. baumannii* AIIMS 7 (biofilm)	16 μg/ml 2 mg/ml	8–12	Singh et al., 2018
*A. baumannii* SRMC 27 (biofilm) *A. baumannii* AIIMS 7 (biofilm)	≤2 mg/ml 25.6 mg/ml	12.05 60	Gaidhani et al., 2013; Salunke et al., 2014
*A. baumannii* ATCC 19606 *A. baumannii* NPRCOE 160575 (MDR)	0.09 μg/ml 0.18 μg/ml	8–15	Wintachai et al., 2019
*A. baumannii* RS307 (carbapenem-resistant)	30 μm^3^	~100	Tiwari et al., 2017
*A. baumannii* NCTC 13305	12.5 μg/ml	10–20	Ebrahimi et al., 2018

1 MIC, minimum inhibitory concentration.

2 Diameter of nanoparticle in nanometres (nm).

3 MIC concentration was reported in μm.

There has been extensive evidence highlighting the synergistic benefits of nanoparticle-antibiotic combination therapies; moreover, many studies have described an enhanced antibacterial effect compared to that of NAg or antibiotics alone (McShan et al., 2015; Baptista et al., 2018; Singh et al., 2018). Some of the general hypotheses behind this synergistic activity suggest that NAg disrupts the bacterial cell envelope and in turn assists in localising antibiotics to their cellular targets, or that NAg conjugates with the biologically active hydroxyl or amino groups present in antibiotics which improves their effective concentration and toxicity (Li et al., 2005; Dakal et al., 2016; Katva et al., 2017). Alternatively, it has been proposed that specific antibiotics can enhance the toxicity of NAg, such as penicillin, by increasing the cell membrane/wall permeability to the nanoparticle (Allahverdiyev et al., 2011; Wan et al., 2016). Wan et al. (2016) observed increasing efficacy of the nanoparticle on *A. baumannii in vitro* when combined with polymyxin-B (PMB), a last-resort membrane permeabilising antibiotic. The study also demonstrated the nanoparticle-antibiotic synergistic effect *in vivo* using *A. baumannii* infected mouse models, increasing mice survival from 0% when treated with PMB (250 μg/kg) alone to 100% when treated with NAg-PMB (2 mg/kg + 50 μg/kg) after a 24 h infection period (Wan et al., 2016). This *in vivo* evidence is important, as it alludes to the therapeutic implications of potential NAg-antibiotic combination treatments for otherwise untreatable bacterial infections (Allahverdiyev et al., 2011; Wan et al., 2016; Baptista et al., 2018). Some other examples of NAg-antibiotic combinations include studies by McShan et al. (2015), who showed a greater extent of MDR *Salmonella typhimurium* growth inhibition with NAg-tetracycline and NAg-neomycin treatments than the nanoparticle alone, and Thomas et al., who reported improved efficacy of various NAg-antibiotic combinations on *S. aureus* and MDR *Staphylococcus epidermidis* (Allahverdiyev et al., 2011; Wan et al., 2016; Baptista et al., 2018).

### Effect of NAg on *A. baumannii* and Other Bacterial Biofilms

The pathogenicity and adaptability of bacteria to antimicrobial agents is significantly attributed to their ability to form biofilms – a surface-attached biological colony made up of one or more bacterial species enclosed by a protective sticky organic matrix called the extracellular polymeric substance (EPS; O’Toole et al., 2000; Donlan, 2002). Biofilms are the predominant mode of growth for over 99% of bacteria, conferring protection against environmental stressors, foreign agents, and toxins, therefore, playing an important role in antibiotic resistance and chronic human infection (Garrett et al., 2008; López et al., 2010; Romanova and Gintsburg, 2011). Antibiotic resistance in biofilms is garnered by several factors, including physical protection by the EPS matrix acting as a diffusion barrier, the stochastic generation of antibiotic tolerant subpopulations (persister cells), the rapid horizontal exchange of genetic material, as well as cell-to-cell communication *via* quorum sensing (Hausner and Wuertz, 1999; Miller and Bassler, 2001; Dufour et al., 2010; Flemming and Wingender, 2010; Lewis, 2010; López et al., 2010). Quorum sensing is a process which allows bacteria to communicate with each other and regulate a range of physiological activities, including conjugation, virulence, and biofilm production (Miller and Bassler, 2001). Biofilm-associated bacteria produce chemical signal molecules called auto inducers which control the expression of these physiological genes (Rutherford and Bassler, 2012).

There is growing attention towards the antibacterial effects of NAg on biofilms. Similar to reports on free-living (planktonic) bacterial systems, many studies have indicated that smaller-sized nanoparticles are more effective at biofilm killing when compared to larger particles, most likely due to the greater SAV and better EPS penetration of the former (see “Size, Shape, and Surface Properties of NAg” section; Choi et al., 2010; Markowska et al., 2013; Martinez-Gutierrez et al., 2013; Radzig et al., 2013). Again, however, it is important to note that the EPS of mature biofilms generally provides colonies with increased protection, rendering them more tolerant to NAg toxicity relative to their planktonic counterparts, as previously seen with *P. aeruginosa* biofilms and other bacterial species (Radzig et al., 2013; Pompilio et al., 2018). Studies have also correlated the anti-biofilm activity of NAg to cellular ROS generation. Qayyum et al. (2017), for example, observed substantial obliteration of *E. coli* and *Streptococcus mutans* biofilms upon exposure to NAg, which was associated with a detected increase in cellular ROS within the biofilm structure, leading to bacterial cell lysis and damage to the protein, polysaccharide, and eDNA constituents of the EPS (Xu et al., 2012; Ezraty et al., 2017).

Studies have also indicated the possible prevention and eradication of biofilm-associated infections with NAg. Wintachai et al. (2019) reported over 90% inhibition of viable MDR *A. baumannii* (NPRCOE 160575) at low NAg doses (0.09 μg/ml or 0.5x the reported MIC), which prevented the attachment and subsequent biofilm formation of (media-suspended) *A. baumannii* on the surface of human lung epithelia (cell line A549). Indeed, the team observed negligible toxicity of the nanoparticle towards to the lung cells (50% cytotoxicity concentration [CC_50_] = 5.72 μg/ml) which is important when considering the medical application of NAg (Wintachai et al., 2019). Singh et al. (2018) reported a greater extent of eradication of *A. baumannii* biofilms with NAg [minimum biofilm eradication concentration (MBEC) = 2 mg/ml] when compared to tetracycline, erythromycin, and doxycycline, citing extensive EPS destruction and reduction in viable cells following nanoparticle treatment. The work also recognised a synergistic effect between erythromycin and NAg against the biofilms, with the antibiotic’s efficacy increasing 32-fold in the presence of the nanoparticle (erythromycin MBEC = 128 mg/ml; erythromycin + NAg MBEC = 4 mg/ml). This again emphasises the potential value of NAg-antibiotic combinations (see “Effect of NAg on *A. baumannii* and other Drug-Resistant Bacteria” section; Singh et al., 2018). Likewise, Qayyum et al. (2017) had shown that catheters coated in NAg hindered the formation of the *E. coli* and *S. mutans* biofilms, which further highlights the healthcare capabilities of the nanoparticle.

Similar to planktonic cells (see “General Antibacterial Mechanisms of NAg (Cell surface)” section), studies have also specified greater Ag^+^ toxicity towards biofilms of various bacterial species when compared to NAg, most likely due to more effective penetration of the ions through the protective EPS layer than the nanoparticles, though there is minimal data on Ag^+^ activity on *A. baumannii* biofilms overall (Radzig et al., 2013; Kędziora et al., 2018). A paper by Vila Domínguez et al. (2020) reported Ag^+^-induced protein damage (thiol group interaction) and DNA condensation on planktonic *A. baumannii* and other bacterial species, leading to subsequent cell death. Vaidya et al. (2017) compared the individual and synergistic efficacy of Ag^+^, gold (Au^+^), copper (Cu^+^), platinum (Pt^2+^), and palladium (Pl^2+^) ions on planktonic and biofilm-forming *A. baumannii* (as well as on *E. faecium* and *K. pneumonia* planktonic and biofilm cells). Ag^+^ and Ag^+^-Cu^+^ were found to be most effective individual and synergistic antibacterial ions against *A. baumannii* biofilms, respectively (Vaidya et al., 2017). Similarly, a study by Shih and Lin (2010) reported the ability of Ag^+^-Cu^+^ to inhibit planktonic and biofilm growth of *A. baumannii* (as well as other bacterial species) in a model plumbing system, providing insights into feasible bacterial biofilm control measures in water distribution systems.

There are a number of other studies that demonstrate effective inhibition and/or eradication of *A. baumannii* biofilms by NAg; however, each use different concentration ranges and methodologies (Salunke et al., 2014; Singh et al., 2016; Ramachandran and Sangeetha, 2017). For example, Salunke et al. (2014) reported ~98% inhibition of *A. baumannii* biofilm formation at very high NAg concentrations (5,120 μg/ml) using a 96-well plate (1,024 μg/200 μl) experimental setup, while Ramachandran and Sangeetha (2017) observed biofilm inhibition at ‘only’ 100 μg/ml concentration of the nanoparticle in a glass test tube setup. Albeit, we should not ignore the fact that these experiments utilised nanoparticles with different physicochemical properties (e.g., NAg sizes of ~64 and ~7 nm, respectively), these inconsistencies in methodology highlight the challenges involved in assessing the antimicrobial efficacy of NAg. Currently, there is no standard protocol to follow for researchers to directly compare the antimicrobial activity of NAg, as the physicochemical characteristics of the nanoparticle, the bacterial growth medium and even the incubation conditions used, would influence its activity (Morones et al., 2005; Loo et al., 2018; Duval et al., 2019).

In summary, NAg has promising potential for prophylactic use and treatment of infections caused by MDR bacteria and their biofilms. The nanoparticle is considered highly effective in inhibiting the colonisation of many antibiotic-resistant bacteria, while also showing strong synergism with conventional antibiotics (Rai et al., 2012; Baptista et al., 2018). However, as previously described in “Introduction” section, advances in nanotechnology have enabled the manipulation and subsequent incorporation of NAg in not only medical devices, but also, increasingly, in everyday arbitrary consumer products (Dakal et al., 2016; Gunawan et al., 2017; Khan et al., 2017). To address this, researchers have investigated the toxic impact of NAg on environmental organisms, plant and animal models, and human cells, with studies still on-going to determine the toxicity threshold and long-term environmental and human health effects of NAg (Burdușel et al., 2018; Ferdous and Nemmar, 2020). The increasingly widespread use of the nanoparticle has also prompted a growing concern over the development of NAg-resistant bacteria, just like in the case of antibiotics, as described henceforth (Graves et al., 2015; Gunawan et al., 2017; Panáček et al., 2018; Valentin et al., 2020).

## Bacterial Adaptations to NAg

Bacterial resistance to silver, specifically to Ag^+^, has been described quite extensively, while evidence of NAg-specific resistance is still emerging. Up to this stage, studies have described the presence of both exogenous and endogenous genetic determinants of Ag^+^ resistance, which is thought to be relevant to NAg also (Gupta et al., 1999; Gunawan et al., 2013; Graves et al., 2015; Panáček et al., 2018; Valentin et al., 2020). The earliest reported case of silver resistance was in an *E. coli* strain isolated from a burn patient in 1969, who was treated with wound dressings coated in 0.5% silver nitrate (AgNO_3_; Jelenko, 1969). Two strains of *Enterobacter cloacae* isolated from a burns ward by Rosenkranz et al. (1974) were found to be resistant to the topical ointment silver sulfadiazine (AgSD). A year later, McHugh et al. (1975) reported on an *S. typhimurium* strain isolated from three burn victims with resistance to AgNO_3_, which also displayed resistance to mercury chloride (HgCl_2_) and various antibiotics.

The molecular basis of silver resistance was first described by Gupta et al. (1999), who discovered an Ag^+^ resistance coding region (*sil* operon) present in a 180 kb plasmid called pMG101. This plasmid was extracted from the silver-resistant *S. typhimurium* strain previously isolated by McHugh et al. (1975) and Gupta et al. (1999). The silver resistance mechanism was first reported by Li et al., who found that the loss of OMPs combined with the upregulation of copper efflux proteins (Cus system) in E. coli conferred resistance to Ag^+^ (Li et al., 1997; Randall et al., 2015). Over the years, several cases of Ag^+^ resistance derived from the Sil/Cus systems (and other mechanisms) have been detected in various bacterial species, including *A. baumannii* (Deshpande et al., 1993; Deshpande and Chopade, 1994; Hosny et al., 2019). Gunawan et al. (2013) were the first to observe NAg resistance in the soil-borne bacterium *B. subtilis* elicited by oxidative-stress mechanisms, which was thought to be associated by the detected presence of *sil* genes. Further studies are published which provide evidence of NAg resistance determinants in different bacteria, which will be described in the following (Graves et al., 2015; Panáček et al., 2018; Valentin et al., 2020).

### Chromosomal (Endogenous) Silver Resistance

**Figure 2:**
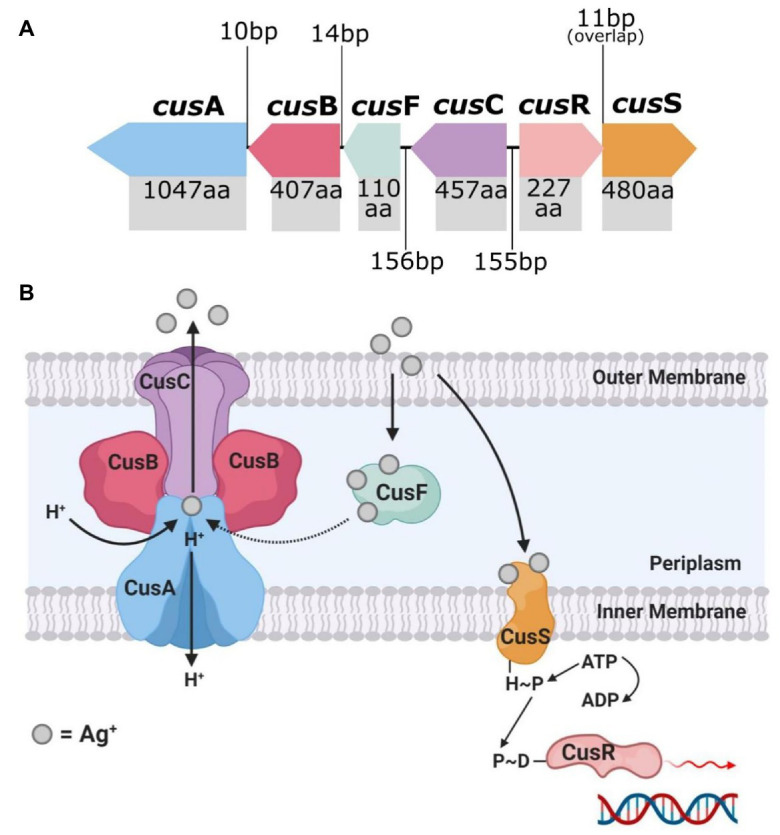
The top image **(A)** shows the genetic arrangement of the *cus* operon and includes the intergenic DNA base pair (bp) gaps/overlaps and each *cus* gene’s protein product amino acid (aa) length. The bottom image **(B)** is a graphical representation of the protein arrangement and functions of the encoded membrane bound Cus efflux system. Created in Inkscape **(A)** and BioRender **(B)**. Adapted from Randall et al. (2015).

The endogenous silver resistance mechanism first discovered in *E. coli* by Li et al. relates to the presence of the Cus efflux system and the loss of major OMPs (Li et al., 1997; Randall et al., 2015). Prolonged exposure of various *E. coli* strains to sub-lethal doses of AgNO_3_ and AgSD (Ag^+^ potent agents) led to the mutational development of Ag^+^ resistance in the bacteria. The mutant Ag^+^-resistant strains displayed a loss of the major porins OmpF or both OmpF and OmpC, along with the expression of a natural copper binding/efflux (Cus) system which conferred cross-resistance to Ag^+^ (Figure 2; Li et al., 1997; Randall et al., 2015; Kędziora et al., 2018). The *cusCFBA* operon is a gene cluster which encodes an active efflux system designed to export copper ions (Cu^+^), and Ag^+^ (Mijnendonckx et al., 2013; Randall et al., 2015). The proteins encoded, CusA, CusB, and CusC, are subunits of a tri-component resistance-nodulation-division (RND)-type efflux system, and CusF, which is periplasmic Ag^+^/Cu^+^ chaperone (Munson et al., 2000; Kędziora et al., 2018). Transcription of *cusCFBA* is regulated by a dual-component system called CusRS (encoded by *cusRS* operon), which sense (CusS) and respond (CusR) to increased levels of Ag^+^/Cu^+^ (Munson et al., 2000; Franke et al., 2003). Exposure of *E. coli* to Ag^+^ can cause a missense mutation in *cusS*, promoting gene transcription and CusS synthesis (and CusR upregulation), where CusS/R then prompts increased expression of *cusCFBA* for active Ag^+^/Cu^+^ binding and efflux (Randall et al., 2015). CusF is a metal-binding chaperone, which binds Ag^+^/Cu^+^ ions to its methionine or cysteine sites and delivers them to CusCBA to be shuttled out of the cell (Lok et al., 2008; Mijnendonckx et al., 2013; Randall et al., 2015). The repression of the major porins OmpF/C in the outer membrane complements the Cus system, as mentioned (Lok et al., 2008; Radzig et al., 2013; Randall et al., 2015). These porins are involved in the transport of cations and small molecules, such as drugs and toxins, across the cell membrane (Koebnik et al., 2000). Transcription of *ompF/C* is regulated by EnvZ/OmpR, whereby EnvZ responds to osmotic changes (i.e., presence of cations) and phosphorylates the transcription unit of OmpR, which then activates the expression of OmpF/C (Cai and Inouye, 2002). Mutation to *envZ*/*ompR* in response to Ag^+^ exposure leads to loss of function to this regulatory system, which results in a reduction of *ompF/C* expression, causing a loss in outer membrane permeability (Li et al., 1997; Lok et al., 2008; Randall et al., 2015). This consequently limits the cytoplasmic access of Ag^+^ and reduces the susceptibility of *E. coli* to Ag^+^ toxicity (Li et al., 1997; Radzig et al., 2013; Randall et al., 2015).

Graves et al. (2015) reported on induced resistance to AgNO_3_ in another *E. coli* strain upon prolonged exposure and, in addition, observed resistance to NAg. The team found non-synonymous point mutations (results in amino acid sequence changes of the protein product) in three genes; *cusS*, as well as *purL*, which encodes the protein phosphoribylsylforml-glycineamide synthetase involved in purine nucleotide biosynthesis, and *rpoB*, which codes for an RNA polymerase beta subunit. As previously discussed, CusS is part of the dual-component sensor/responder regulator for the CusCFBA Ag^+^/Cu^+^ efflux system (Franke et al., 2003; Lok et al., 2008). Resistance to Ag^+^ in the *E. coli* strain was significant and defined, with a >26-fold increase in Ag^+^ concentration at which the resistant bacterium could proliferate (compared to the wild-type strain), while the NAg-resistant strains could grow at a lower 1.4–4.7-fold increase in dose. It was unclear as to what exact mechanisms conferred the observed resistance to NAg in *E. coli*. As the leaching of Ag^+^ from NAg is an integral part of the nanoparticles antibacterial activity, it is thought that the CusCFBA Ag^+^ efflux system played some role in the NAg resistance effect (Graves et al., 2015).

According to Randall et al. (2015), the Cus system is not unique to the *E. coli* genome and has been found in strains of other bacteria, including the soil and human gut bacterium, *Citrobacter freundii*, and the pathogenic gut bacterium, *Shigella sonnei*. However, no changes in OMP expression in these bacteria (no loss of the porins) were observed, which could perhaps explain their susceptibility to Ag^+^, suggesting that the Cus system alone is not sufficient for a bacterium to confer resistance to silver (Randall et al., 2015). The *cus* operon and OMP mechanisms are specific to Gram-negative bacteria; however, silver resistance, to both Ag^+^ and NAg, has also been observed in Gram-positive bacteria. Apart from the original discovery in *B. subtilis* by Gunawan et al., studies have also detected resistance in the clinically-relevant species, *S. aureus* (see “Other Mechanisms of Silver Resistance against NA” section), and silver resistance in these bacteria has been associated with *sil* genes and mutations of physiological genes (Loh et al., 2009; Randall et al., 2013; Valentin et al., 2020).

Resistance to Ag^+^ has been previously reported in *A. baumannii*, which will be discussed further; however, no endogenous silver resistance mechanisms have been detected to date in this bacterium (Deshpande and Chopade, 1994; Shakibaie et al., 2003; Hosny et al., 2019). Alquethamy et al. (2019) provided the first report of a highly conserved chromosomally-encoded copper resistance system in *A. baumannii* which was distinct from the other known copper resistance mechanisms, including the Cus system. The mechanism involves two transcriptional regulators of copper-resistance, CueR and CopRS, as well as a P-type ATPase Cu^+^ efflux protein called CopA (Williams et al., 2016; Alquethamy et al., 2019). The existence of this conserved chromosomal Cu^+^ efflux system suggests that a mutational response to copper exposure may have occurred at some point earlier in the phylogeny of *A. baumannii* and has been maintained through natural selection. The copper and silver efflux mechanisms, i.e., Cus and Sil systems, are homologous as they share common protein sequences and elicit copper/silver cross-resistance in bacteria; therefore, it is possible that the CueR/CopRS/CopA could also be involved in a silver resistance effect in *A. baumannii*.

### Plasmid-Mediated (Exogenous) Silver Resistance

**Figure 3:**
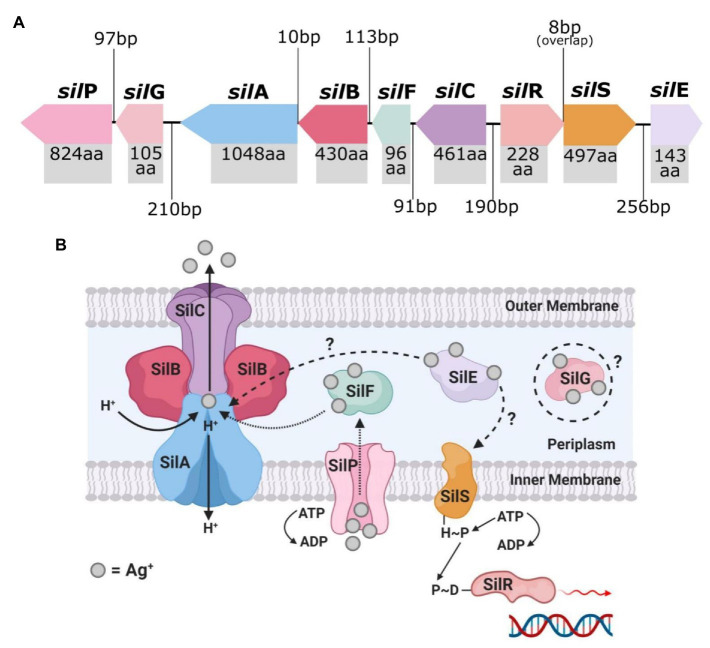
The top image **(A)** shows the genetic arrangement of the *sil* operon and includes the intergenic DNA base pair (bp) gaps/overlaps and each *sil* gene’s protein product amino acid (aa) length. The bottom image **(B)** is a graphical representation of the known and predicted protein arrangement and functions of the encoded membrane bound Sil efflux system. Created in Inkscape **(A)** and BioRender **(B)**. Adapted from Randall et al. (2015).

The Ag^+^-resistant *S. typhimurium* strain isolated by McHugh et al. (1975) is the source of the most cited and researched silver resistance mechanism to date. In 1999, Gupta et al. isolated the HI-2 incompatibility group (IncHI-2) plasmid pMG101 from this bacterium and found that it contained various genes that encode resistance to several heavy metals and antibiotics (Gupta et al., 1999, 2001). The plasmid segment conferring silver resistance determinants contained the *sil* operon, which consists of nine genes that encode a Ag^+^ binding and efflux system (Figure 3; Gupta et al., 1999; Silver, 2003; Niño-Martínez et al., 2019). In reading order, these genes are *silE*, *silS*, *silR*, *silC*, *silF*, *silB*, *silA*, *ORF105 (silG)*, *and silP*, and encode their proteins in three transcriptional units (SilE, SilRS, and SilCFBAGP; Silver, 2003; Andrade et al., 2018). The characterisation and organisation of the Sil system are in fact based on the earlier discovered Cus system, as the two efflux systems share close peptide homologies, as mentioned previously (Mijnendonckx et al., 2013; Randall et al., 2015; Kędziora et al., 2018). However, in contrast to the Cus system, the exogenous Sil system does not associate with the loss of OMP porins (i.e., OmpF/C) to evoke Ag^+^ resistance (Randall et al., 2015).

SilE is a periplasmic protein chaperone that binds to free Ag^+^ present in the periplasm and is currently the only Sil protein which has had its function fully validated (Gupta et al., 1999; Randall et al., 2015). SilRS are dual-component Ag^+^ sensor (SilS) and responder (SilR) transcriptional regulators for SilCFBAGP, and are direct homologs of CusRS (Silver, 2003; Kędziora et al., 2018). SilCFBA shares ~80% protein homology with CusCFBA and functions as a multi-component RND-type Ag^+^ efflux system (Randall et al., 2015; Kędziora et al., 2018). SilA is an inner membrane cation/proton efflux antiporter, SilB functions as membrane fusion protein which clamps SilA together with SilC, an OMP channel (Andrade et al., 2018). SilF is another periplasmic Ag^+^-binding chaperone (similar to SilE), and binds with Ag^+^ that have passed through the outer membrane from outside the cell (Kędziora et al., 2018). SilP is an inner membrane-bound P-type ATPase efflux which transports Ag^+^ from the cytoplasm to the periplasm for binding with the chaperones SilE/F (Mijnendonckx et al., 2013). The *sil* operon also contains an unspecific open-reading frame *ORF105* which codes for a protein with currently no defined function (Gupta et al., 1999; Mijnendonckx et al., 2013; Andrade et al., 2018). The encoded protein shares a ~45% amino acid sequence homology with the Cu^+^ chaperone CopG, and both contain a CXXC motif (two amino residues between two cysteine), which is known to be involved in heavy metal binding (Su et al., 2007; Randall et al., 2015; Andrade et al., 2018). To fit the *sil* gene nomenclature, Randall et al. (2015) proposed that *ORF105* be given the name *silG* and described as an Ag^+^-binding chaperone, similar to SilE/F, until proven otherwise.

### Presence of Sil System in *A. baumannii* and Other Species

The *sil* operon has been found in other IncHI plasmids like pMG101 (Table 2), and due to horizontal gene transfer of these plasmids between bacteria, whole or part of the operon has been detected among many bacterial species (Gupta et al., 2001; Mijnendonckx et al., 2013). Since the initial discovery of *sil* genes in *S. typhimurium*, studies have further detected the genes in other *Salmonella* spp., and in other Gram-negative bacteria, including *E. cloacae*, *E. coli*, *P. aeruginosa*, *K. pneumoniae*, *Serratia marcescens*, and *A. baumannii*, as well as Gram-positive bacteria, such as *B. subtilis* and *S. aureus* (Deshpande and Chopade, 1994; Gunawan et al., 2013; Finley et al., 2015; Hosny et al., 2019; Valentin et al., 2020). Hosny et al. (2019) discovered *sil* operon-harbouring plasmids in various clinical species isolated from burns/wounds of hospital patients, including two MDR *A. baumannii* strains. Conjugative horizonal transfer of the *sil* operon was observed between the silver-resistant *A. baumannii* strains and non-silver-resistant *E. coli*. This resulted in the expression of the *sil* genes and subsequent development of a Ag^+^ resistance phenotype in the latter bacterium (Hosny et al., 2019). Deshpande and Chopade (1994) had in fact reported the conjugal transfer of silver resistance determinants many years prior. Their work observed the transfer of plasmid pUPI199, which contained undefined silver resistance genes, from an *A. baumannii* strain (BL88) to *E. coli* with, once again, subsequent expression of the silver resistance phenotype (Deshpande and Chopade, 1994). A study by Shakibaie et al. (2003) also detected the presence of another plasmid-associated silver resistance mechanism in an *A. baumannii* strain (BL54), which is unrelated to the Sil system, and was thought to involve in the intracellular accumulation of Ag^+^ and binding of the ions to metalloproteins to form inert silver complexes. No follow-up inquiries have been made on either of these non-*sil* derived silver resistance determinants, and, therefore, it is difficult to assess the significance of these resistance mechanisms.

**Table 2:** Properties of known HI incompatibility group (IncHI) plasmids containing genes coding for silver resistance, including either the complete *sil* operon or some *sil* genes.

Genus/species	Plasmid	*sil* genes	Size (bp)	Conjugative	GenBank acc. no.	Reference
*S. typhimurium*	pMG101	*ESRCABGP*	14,211	Y	AF067954	Gupta et al., 1999
*Serratia marcescens*	R476b	*E* *P* *S*	424 1,362 1,154	Y	AY009372 AH011380 AH011381	Gupta et al., 2001
*Salmonella enterica*	MIP233	*E* *P* *S*	424 1,356 1,154	Y	AY009382 AH011384 AH011385	Gupta et al., 2001
*S. enterica*	pWR23	*E* *P* *S*	424 1,356 1,154	Y	AY009387 AH011388 AH011389	Gupta et al., 2001
*S. enterica*	MIP235	*E* *P* *S*	424 1,356 1,154	Y	AY009392 AH011386 AH011387	Gupta et al., 2001
*S. marcescens*	R478	*ESRCABGP*	274,762	Y	BX664015	Gilmour et al., 2004
*E. coli*	pAPEC-O1-R	*ESRCABGP*	241,387	Y	DQ517526	Johnson et al., 2006
*Salmonella typhi*	R27	*ESRCABGP*	180,461	Y	AF250878	Sherburne et al., 2000
*A. baumannii*	pUPI199	nk^1^	~50,000	Y	nk^2^	Deshpande and Chopade, 1994

1 Unknown if detected silver resistance genes are sil genes.

2 Accession number could not be found in GenBank.

### Other Mechanisms of Silver Resistance Against NAg

Most studies have established that bacterial resistance to silver can develop through genetic mutations, as well as through horizontal gene transfer (i.e., *via* plasmids; see “Chromosomal (Endogenous) Silver Resistance” and “Plasmid-mediated (Exogenous) Silver Resistance” sections). However, there is evidence that an increase in expression of native bacterial processes can also contribute to silver resistance. For example, a study by Muller et al. outlined that the redox-active metabolite pyocyanin, produced by *P. aeruginosa*, could reduce extracellular Ag^+^ to non-toxic Ag^0^ and, in turn, confer resistance to the ions (Muller and Merrett, 2014). Another example was the increased production of EPS by planktonic *E. coli*, which acted as a permeability barrier to Ag^+^, causing neutralisation and agglomeration of the ion into inert particulates (Kang et al., 2013). A study by Panáček et al. (2018) revealed an intrinsic NAg resistance mechanism in *E. coli* involving overproduction of the protein flagellin, which led to the aggregation of the nanoparticles. Flagellin is an adhesive protein, forming part of the structural component in the bacterial motility organelle flagella and is known to be involved in biofilm formation (Metlina, 2004; Lu and Swartz, 2016; Panáček et al., 2018). This resistance mechanism was considered epigenetic, as it was independent of any genetic mutations, and provided no observable resistance to Ag^+^ due to the solubility of the ions (Panáček et al., 2018).

As mentioned in “Chromosomal (Endogenous) Silver Resistance” section, evidence of silver resistance in Gram-positive bacteria have been reported, although less frequent in comparison to Gram-negative bacteria. Loh et al. (2009) examined the frequency of *sil* gene occurrences in 36 *S. aureus* strains isolated from human and animal sources. Three strains were found to contain only the *silE* gene (95–100% homology with *silE* in pMG101), which appeared to confer transient resistance upon exposure to Ag^+^ through ion binding; however, the exposure eventually resulted in cell death (Loh et al., 2009). In another study, however, Hosny et al. (2019) isolated four clinical MDR *S. aureus* strains, each displaying stable resistance to Ag^+^, and found one strain expressed the complete *sil* operon, while the remaining three expressed some of the *sil* genes. The study on *B. subtilis* by Gunawan et al. (2013) is the only other research inquiry apart from that of Valentin et al. (2020) that reported the development of NAg resistance in Gram-positive bacteria. Valentin et al. (2020) showed the development of stable resistance to NAg (and Ag^+^) in *S. aureus* (ATCC 25923) through prolonged exposure, with no known presence of the *sil* genes in its genomes. The bacterium developed physiological genetic mutations, the first reported case of NAg-induced single nucleotide polymorphisms in a Gram-positive bacterium (Valentin et al., 2020). More specifically, mutations in *purR*, which encodes a purine repressor regulator protein, were hypothesised to lead to an upregulation in purine nucleotide synthesis to cope with DNA targeted NAg activity. The study also detected mutations in *tcyA*, which codes for an L-cystine binding protein, lowering the influx of extracellular cystine which helped reduce oxidative stress by ROS generated from both high levels of intracellular cysteine and by NAg and Ag^+^ (Park and Imlay, 2003; Sinha et al., 2003; Valentin et al., 2020).

### Silver and Other Metals as Drivers of Antibiotic Resistance

**Figure 4:**
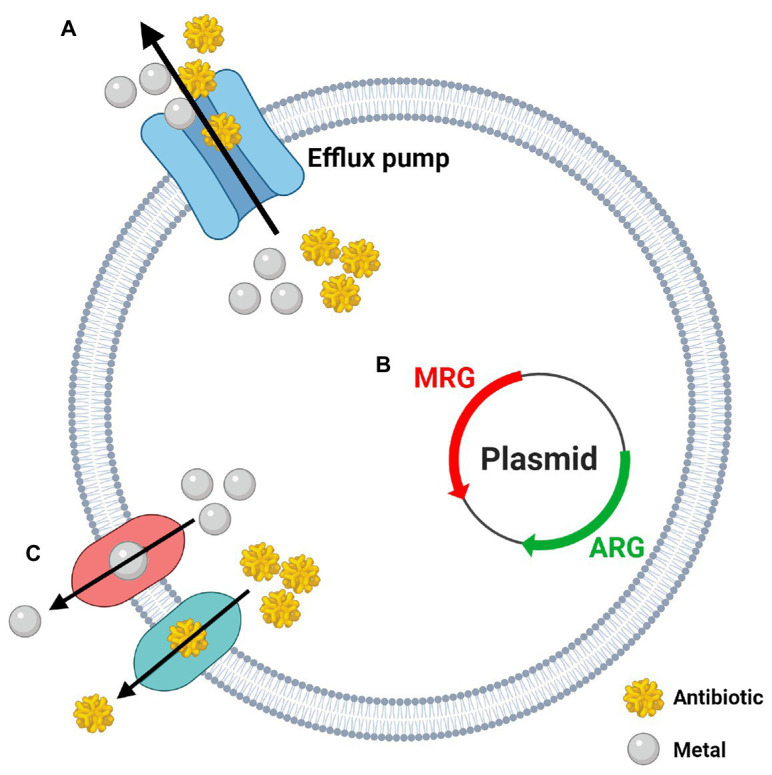
The three potential mechanisms behind the co-selection of heavy metal and antibiotic resistance. **(A)** Cross-resistance: one gene/mechanism confers resistance to metals and antibiotics at once (i.e., efflux pumps); **(B)** Co-resistance: genes coding for metal resistance (MRG) and antibiotic resistance (ARG) are grouped together on the same genetic element (i.e., plasmids); **(C)** Co-regulation: expression of individual metal and antibiotic resistance systems are managed by a common gene or regulator. Created in BioRender. Adapted from Pal et al. (2017; Lic. No. 4986171126814).

While bacterial resistance to silver (NAg and Ag^+^) is itself a troubling issue, there has been emerging evidence to show that silver and other heavy metals (e.g., lead, cadmium, chromium, mercury, etc.) can co-select for antibiotic resistance (Ma et al., 2016; Siddiqui et al., 2019). The emergence of heavy metal/antibiotic resistance was first described in 1974, when Koditschek and Guyre (1974) isolated *E. coli* from a sludge-contaminated estuarine and found it had “indirectly” acquired antibiotic resistance due to heavy metal exposure. Agriculture and aquaculture practices across the globe frequently use metal-containing fertilisers, pesticides, and feed additives, and have consequently contributed to profound environmental accumulation of heavy metals. Heavy metals are stable and are not subject to rapid degradation, thus their presence in soil and water is thought to be significantly greater than antibiotics, and may, therefore, contribute to long-term exposure and selective pressure on bacteria (Baker-Austin et al., 2006; Seiler and Berendonk, 2012). The co-selection of antibiotic resistance by heavy metals is often associated with ‘dual’ metal/antibiotic resistance phenomena – cross-resistance, co-resistance, or co-regulation/co-expression (Figure 4; Baker-Austin et al., 2006). Cross-resistance occurs when the same genes encode resistance mechanisms to multiple agents, and in the case of metal/antibiotic resistance, one key example is the expression of Tet efflux pumps, which export tetracyclines and zinc ions (Zn^2+^; Chapman, 2003). Co-resistance arises when different genes present in the same genetic element confer resistance to multiple agents at once, and are often found in mobile genetic elements (plasmids, transposons, or integrons; Baker-Austin et al., 2006). As an example, plasmid pMG101, from which the *sil* genes were first discovered, was found to also contain resistance genes for mercury and tellurite, and for a number of antibiotics, including ampicillin and chloramphenicol (Gupta et al., 1999). Likewise, plasmid pUPI199 isolated from *A. baumannii* by Deshpande and Chopade (1994), which harboured (undefined) silver resistance genes, had also been found to contain resistance genes for 12 other metals and 10 antibiotics. Co-regulation is when regulatory genes (in a chromosome or plasmid) of different resistance mechanisms are transcriptionally linked, meaning, in this case, exposure to a metal can trigger the expression of both metal resistance genes (MRGs) and antibiotic resistance genes (ARGs; Baker-Austin et al., 2006). For example, overexpression of the gene *robA* [encodes the right origin-binding (Rob) transcriptional regulator protein] in *E. coli* was found to activate multiple mechanisms which gave rise to resistance phenotypes to silver, mercury, and cadmium, as well as various antibiotics and organic solvents (Nakajima et al., 1995).

A recent paper by Siddiqui et al. (2019) reported an increased co-selection of extended-spectrum beta lactamases (ESBLs; confer resistance to β-lactam antibiotics such as penicillins, carbapenems, cephalosporins, etc.) and silver resistance determinants (*sil* genes) in the polluted Yamuna River in India. From the collected bacterial isolates, 121 were found to be ESBL producers, including various *Acinetobacter*, *Enterobacteriaceae*, and *Bacillus* species, with the most prevalent ESBL gene being *bla*_CTX-M_ (encodes class A β-lactamases; commonly targets cephalosporins, i.e., cefotaxime). Out of the 73 isolates containing *bla*_CTX-M_ genes, 53 were found to have at least one *sil* gene (*silE*, *silP*, or *silS*), and worryingly, the most common ‘dual’ presence of ESBL/*sil* genes in these isolates were *bla*_CTX-M_ + *bla*_TEM_ (encodes other class A β-lactamases; commonly target penicillins) + *silE* + *silP* + *silS*. All ESBLs and *sil* genes were present in plasmids, and this work found these resistance genes could be horizontally transferred to a plasmid-free *E. coli*, rendering the bacterium resistant to β-lactams (including penicillins and cephalosporins) and Ag^+^, confirming the co-resistance effect (Siddiqui et al., 2019). Another study by Deshpande et al. (1993) described a correlation between β-lactamase production and metal ion resistance in various clinical strains of *A. baumannii* and other *Acinetobacter* spp. The bacterial strains that were sensitive to toxic metals (including silver, mercury, and cadmium) were associated with lower levels β-lactamase gene expression, while those that were resistant to the metals were associated with higher expression levels of the ARGs. This correlation was particularly evident in *A. baumannii* (Deshpande et al., 1993; Abdar et al., 2019). Deshpande et al. suggested that the resistant *A. baumannii* strains were carrying plasmids that contained both β-lactamase and MRGs (Deshpande et al., 1993; Deshpande and Chopade, 1994; Veress et al., 2020). Other bacterial species have also been found to carry plasmids that harbour *sil* genes and ARGs. For example, Gilmour et al. (2004) found that the plasmid R478 isolated from the opportunistic pathogenic bacterium *S. marcescens* carried the entire *sil* operon, along with mercury, tellurite, and copper resistance genes, as well as tetracycline, chloramphenicol, and kanamycin resistance genes. Similarly, Johnson et al. (2006) also found the entire *sil* operon in plasmid pAPEC-O1-R isolated from *E. coli*, which also carried copper resistance genes and several ARGs, including gentamicin, streptomycin, and tetracycline resistance genes. Most antibiotic-resistant *A. baumannii* strains belong to the GC1 complex, many of which carry a large resistance gene island (AbaR) which contains several ARGs, including ESBLs (e.g., *bla*_TEM_), and MRGs for mercury, cadmium, and zinc, and could perhaps alternatively explain the observations made by Deshpande et al. (1993) and Hamidian and Hall (2018). The high incidence of infections by MDR *A. baumannii* throughout the Iraqi-Afghan conflict is one of the most important examples regarding heavy metal-driven antibiotic resistance co-selection in this species (Howard et al., 2012). Destroyed infrastructure and metal-based military equipment (i.e., munitions, ordnance, and explosives) are known to contaminate environments through heavy metal leaching (Gębka et al., 2016; Vänskä et al., 2019). It has been proposed that metal exposure on *A. baumannii* in contaminated soil or water, for example, could have promoted the co-selection of antibiotic resistance, which resulted in the increased prevalence of MDR-resistant *A. baumannii* infections in soldiers exposed to these environments during combat (Bazzi et al., 2020).

Silver nanoparticle has also been shown to promote the co-emergence of antibiotic resistance in bacteria (Ma et al., 2016; Chen et al., 2019b; Pietsch et al., 2020). A study by Ma et al. (2016) found that treatment of waste water with NAg (and Ag^+^) using lab-scale sequencing batch reactors (SBRs), resulted in an increased shift in various ARGs among isolated Burkholderia spp., Streptomyces spp., and Gemmatimonas spp. More specifically, metagenomics data associated the detection of HAE1 family protein (multi-drug efflux protein), *strA* gene (encodes aminoglycoside 3'-phosphotransferase; aminoglycoside resistance), and *acrB* gene (encodes multidrug efflux pump subunit AcrB) with the increased presence of NAg, while increased abundance of undecaprenol kinase and undecaprenyl-disphosphatase (resistance to bacitracin) and *ermF* gene (encodes rRNA adenine N-6-methyltransferase; macrolide resistance) was associated with Ag^+^ presence (Ma et al., 2016). Interestingly, the team also found that the total abundance of MRGs (including *sil* genes) were highest in the SBRs treated with NAg, suggesting that the nanoparticle has the most potential for ARG co-selection compared to Ag^+^ (Ma et al., 2016). There is also evidence to show that the formation of biofilms functions as a mechanism for co-selecting antibiotic and metal resistance (Baker-Austin et al., 2006; Pietsch et al., 2020). The EPS matrix of biofilms acts as a sequestering barrier to heavy metals and antibiotics, and alarmingly, studies have found that exposure of metals on biofilms can encourage EPS synthesis, improving the biofilms adhesive, structural, and protective integrity (Yang and Alvarez, 2015; Song et al., 2016). Moreover, because of the proximity of biofilm-cells, there is a much greater magnitude of DNA conjugation between bacteria. Research shows that these events can increase under stressful conditions (i.e., exposure to antibacterial agents), which is advantageous to the co-selection process (Song et al., 2016; Singh et al., 2017). Studies have also found that exposure of NAg/Ag^+^, and other heavy metal agents, on biofilms can stimulate quorum sensing, increasing expression of genes involved in biofilm formation and conjugation of ARGs (Qiu et al., 2015; Yang and Alvarez, 2015). Yang and Alvarez (2015) revealed that the treatment of *P. aeruginosa* biofilms with sub-lethal doses of NAg stimulated an upregulation in quorum sensing, resulting in increased EPS production and subsequent biofilm formation. They also found it induced an up to 3.4-fold increase in expression of the multi-drug efflux gene *mexA* (Yang and Alvarez, 2015). The regulation and promotion of biofilm formation and change in bacterial gene expression (including lateral transfer of ARGs) have been observed in *A. baumannii* in response to cationic iron (Fe^+^) exposure previously, for example, but not to silver (NAg or Ag^+^; Qiu et al., 2012, 2015).

While bacteria have been exposed to toxic heavy metals long before human existence, anthropogenic pollution of environments has evidently created prolonged selective pressures on bacteria, consequently promoting the co-emergence of heavy metal and antibiotic resistance (Baker-Austin et al., 2006). It is, therefore, critical to recognise the implications heavy metals (including silver) have on bacteria in both the environment and in clinical settings, as the co-selection of ARGs and promotion of biofilm growth could further strain the healthcare system and exacerbate the current drug resistance crisis.

## Knowledge Gap and Final Remarks

Global antibiotic resistance is not a future threat, but one that has at last transpired. From improper prescriptions for non-bacterial infections and overuse in agriculture/food industries, to the diminishing pharmaceutical investment into their development, the misuse of antibiotics ultimately calls for the need of novel and alternative antibacterial agents (Ventola, 2015; World Health Organization, 2017; Centers for Disease Control and Prevention, 2019). Major advancement in nanotechnology has led to significant progress in designing many antibacterial nanoparticles. The metal-based silver nanoparticle (NAg) is currently the most developed nanoparticle due to its multi-targeting antibacterial mechanisms and proven efficacy against a broad-spectrum of bacteria (Silva, 2004). Many studies have shown that NAg is highly toxic to several Gram-positive and Gram-negative bacterial species, including the ESKAPE pathogens – a consortium of bacteria that frequently exhibit multi-drug resistance and are the leading cause of nosocomial (hospital-related) infection (Rai et al., 2012). Among this group (carbapenem-resistant), *A. baumannii* is of key concern, having been recently declared the “number one” critical level priority pathogen. Thus calling for the immediate development of alternative antibacterial treatments for this highly infectious and resistant pathogen (Gonzalez-Villoria and Valverde-Garduno, 2016; Hamidian and Nigro, 2019). *A. baumannii* and other globally prevalent pathogens have become the main targets of the unique and effective antibacterial nanoparticle.

Notwithstanding the strong antibacterial efficacy of NAg, there has been a growing concern over the ability of bacteria to adapt to the nanoparticle due to its increasingly widespread use (Gunawan et al., 2017). Bacterial resistance to the ionic form of silver, Ag^+^, has been recognised for many years, and in the last decade, research inquiries have indeed observed the development of resistance mechanisms against NAg in several environmental and clinically-relevant Gram-negative and Gram-positive bacteria (Gupta et al., 1999; Gunawan et al., 2013; Graves et al., 2015). Further, studies have shown that biofilms – a resilient surface-attached bacterial community, can also adapt to silver (both NAg and Ag^+^) despite the effective biofilm inhibiting and eradicating activity of the silver agents compared to many conventional antibiotics (Radzig et al., 2013; Yang and Alvarez, 2015). Biofilms are a major healthcare issue, as they frequently develop in cases of uncontrolled and chronic infections (López et al., 2010).

To the best of our knowledge, only the planktonic form of *A. baumannii* has been found to exhibit Ag^+^ resistance characteristics due to the presence of the exogenous (plasmid-based) Ag^+^ efflux Sil system, and, as found in some cases, other undefined/non-Sil related mechanisms (Deshpande and Chopade, 1994; Hosny et al., 2019). No presence of endogenous (chromosomal) silver resistance mechanisms have been identified in *A. baumannii*, so far. However, we are not ignoring the fact that chromosomally encoded copper efflux systems have been detected in this bacterium, which could infer the possibility of chromosomal silver resistance due to the similarities between copper and silver efflux mechanisms (Williams et al., 2016; Alquethamy et al., 2019). To date, no work has been undertaken to determine if *A. baumannii* can develop resistance or specific adaptation(s) to NAg exposure. Our team is currently studying the nanoparticles toxicity on *A. baumannii* in both its planktonic and dominant biofilm form of growth, and in turn, the adaptation characteristic(s) of the bacterium which could develop in response to prolonged exposure. While it is possible that the Sil system plays a role in resistance to NAg, given that the nanoparticle exerts its toxicity differently from Ag^+^, it is likely that resistance to NAg involves additional mechanisms that are still largely unexplored.

In addition, evidence has emerged showcasing the potential for heavy metals to co-select for antibiotic resistance genes (ARGs), facilitated by ‘dual’ metal/antibiotic resistance mechanisms which are related to cross-resistance (same genes/mechanism conferring resistance to both metal and antibiotics), co-resistance (metal and antibiotic resistance determinants located in the same genetic element), or co-regulation (the regulatory genes of metal and antibiotic resistance determinants are transcriptionally linked; Baker-Austin et al., 2006). The combined effects of heavy metal exposure on bacteria in potentially driving both metal resistance and antibiotic resistance is a troubling issue, and further highlights the important implications of heavy metal overexposure on bacteria and to subsequently minimise this risk. Both NAg and Ag^+^ have been found to facilitate the co-selection of various ARGs, most frequently seen at this stage in polluted water systems (Ma et al., 2016; Siddiqui et al., 2019). Over the years, studies have indeed shown that *Acinetobacter* spp. (including *A. baumannii*) can display cross-resistance to Ag^+^ (and other heavy metals) and several antibiotics, but there is yet to be any evidence of NAg-induced co-selection of ARGs in this bacterial genus.

The increasing prevalence of silver resistance combined with growing evidence of the co-emergence of heavy metal and antibiotic resistance highlights the serious issues behind antibacterial overuse. Our knowledge of bacterial resistance to silver and other heavy metals will help equip us to study the complex adaptation mechanisms of bacteria against NAg. Elucidating the mechanisms of NAg resistance could enable the development of technologies that mitigate these problematic adaptation responses. The generated knowledge of how the nanoparticle targets bacteria, and, in turn, how bacteria develop responses to its multi-targeting mechanisms can help guide the physicochemical engineering process of NAg (e.g., morphology, oxidation state, and surface charge) to fine tune its antibacterial activity and, therefore, limit bacterial adaptation. Identification of the molecular basis of NAg resistance will allow us to target the biological signalling molecules and metabolites that trigger adaptation responses, including quorum sensing molecules and/or epigenetic and genetic regulators. The generated knowledge will also help inform strategies for a better risk vs. benefits assessment regarding the application of NAg-containing consumer products, limiting its misuse and inadequate disposal. With no discovery of new effective antibiotics over the last 30 years, we need to protect the efficacy of this valuable alternative antimicrobial agent so that we may continue to use it in the fight against untreatable infections.

## Author Contributions

OM, MH, and CG: conceptualisation. OM: writing (original draft preparation). OM, RM, MH, and CG: writing (review and editing). MH and CG: supervision. All authors have read and agreed to the published version of the manuscript.

### Conflict of Interest

The authors declare that the research was conducted in the absence of any commercial or financial relationships that could be construed as a potential conflict of interest.
